# Feasibility, Technical Safety, and Early Clinical Experience with an Automated Annular Suturing Device in Minimally Invasive Valve Replacement

**DOI:** 10.3390/jcm15176870

**Published:** 2026-09-04

**Authors:** Robert Balan, Parwis Massoudy, Marius Mihai Harpa, Klara Brînzaniuc, Jonah Schwarz

**Affiliations:** 1 Doctoral School, George Emil Palade University of Medicine, Pharmacy, Science and Technology of Targu Mures, 540142 Targu Mures, Romania; 2 Department of Cardiac Surgery, Klinikum Passau, 94032 Passau, Germany; parwis.massoudy@klinikum-passau.de (P.M.); jonah.schwarz@klinikum-passau.de (J.S.); 3 Department of Surgery IV, George Emil Palade University of Medicine, Pharmacy, Science and Technology of Targu Mures, 540142 Targu Mures, Romania; marius.harpa@umfst.ro; 4 Department of Cardiovascular Surgery, Emergency Institute for Cardiovascular Diseases and Transplantation Targu Mures, 540136 Targu Mures, Romania; 5 Department of Anatomy and Embryology, George Emil Palade University of Medicine, Pharmacy, Science and Technology of Targu Mures, 540142 Targu Mures, Romania; klara.brinzaniuc@umfst.ro

**Keywords:** minimally invasive surgery, percutaneous cannulation, mitral valve surgery

## Abstract

**Background:** Minimally invasive cardiac valve surgery (MIVS) provides distinct clinical advantages, yet annular suturing remains a technically demanding step. Automated annular suturing devices (RAM^®^) have been developed to standardize suture placement during valve replacement. This study evaluates the initial clinical feasibility, technical safety, and operational integration of automated suturing during its institutional adoption phase. **Methods:** We retrospectively evaluated 43 consecutive patients undergoing MIVS supported by the RAM^®^ system (2021–2025), stratified into mitral valve replacements (RAM-MVR, *n* = 26) and aortic valve replacements (RAM-AVR, *n* = 17). Technical parameters, procedural safety metrics, and perioperative outcomes were descriptively analyzed and contextualized against our broader institutional MIVS platform using percutaneous femoral cannulation (*n* = 182). **Results:** Automated annular suturing achieved a 100% technical feasibility rate (43/43, 95% CI: 91.8–100.0%) with zero mechanical device failures or intraoperative suture-line disruptions (0%, 95% CI: 0.0–8.2%). In the RAM-MVR cohort, median cross-clamp and cardiopulmonary bypass (CPB) times were 73.0 min (IQR 64.0–91.0) and 114.5 min (IQR 94.8–129.5), respectively. In RAM-AVR, median cross-clamp time was 75.0 min (IQR 71.0–83.0). Thirty-day mortality was 4.7% (2/43, 95% CI: 0.6–15.8%), secondary to non-device-related medical complications in high-risk baseline patients. Postoperative echocardiography demonstrated no paravalvular regurgitation (0%, 95% CI: 0.0–8.2%). **Conclusions:** Initial institutional experience demonstrates that automated annular suturing is technically feasible and safe in minimally invasive mitral and aortic valve replacement. The system enables consistent procedural execution across valve positions. These observational data support automated suturing as a feasible alternative to manual suturing in MIVS workflows.

## 1. Introduction

Minimally invasive cardiac valve surgery (MIVS) has evolved over the past two decades into a well-established and robust alternative to conventional median sternotomy for selected patients requiring mitral or aortic valve interventions [1,2,3,4,5]. Since the pioneering work of Cohn [1], which demonstrated that limited-access valve surgery significantly improved patient satisfaction and reduced costs, the field has transitioned toward increasingly sophisticated techniques. Numerous systematic reviews, meta-analyses, and large-scale registry studies have confirmed that minimally invasive approaches achieve clinical outcomes and safety profiles comparable to conventional surgery while offering distinct physiological advantages [6,7]. These benefits include reduced surgical trauma, shorter hospital stays, accelerated postoperative recovery, and superior cosmetic results. Consequently, MIVS has been increasingly adopted as the standard of care for both isolated and selected combined valve procedures in high-volume centers, or even in patients with thoracic malformations [8].

Despite these clear clinical advantages, MIVS remains technically demanding and the reliance on restricted access, deep operative fields, and long-shafted instrumentation—often coupled with endoscopic visualization—substantially increases procedural complexity [3]. These factors place high ergonomic and cognitive demands on the surgical team, which can impact workflow efficiency. Challenges are most pronounced during the annular suturing phase, a step that requires absolute precision in needle placement and controlled tissue handling within confined anatomical spaces. Unlike conventional surgery, where the surgeon has direct 3D visualization and manual dexterity, MIVS often necessitates specialized suturing techniques to ensure the long-term stability of the prosthesis and to prevent paravalvular leaks [4]. Because of these constraints, prolonged cardiopulmonary bypass (CPB) and aortic cross-clamp times are frequently reported, particularly during the developmental phase of a minimally invasive program [9,10,11,12], alongside procedure-specific perioperative challenges associated with lateral mini-thoracotomy and altered pulmonary mechanics [13].

The clinical implications of operative duration cannot be overstated. Prolonged ischemic and bypass times are major concerns in cardiac surgery, as aortic cross-clamp duration has been identified as an independent predictor of morbidity and mortality across diverse patient populations [9,10,11,12]. Strategies aimed at stabilizing or reducing these operative times without compromising procedural safety or repair quality are therefore of paramount interest. In this context, technological innovation has played a central role in mitigating the technical hurdles of limited-access surgery. For instance, automated knot-fastening devices have been widely adopted in both minimally invasive and robotic valve surgery, with several meta-analyses associating their use with significant reductions in cross-clamp and CPB times [7,14,15,16]. Building upon the success of mechanized knot-tying, automated annular suturing devices—such as the RAM^®^ system (LSI Solutions, Victor, NY, USA)—have been developed to further mechanize the procedure by automating the placement of the annular sutures themselves [15,17].

The RAM^®^ device is designed to improve reproducibility, reduce ergonomic strain, and enhance sharps safety by automating a traditionally manual and high-risk step. Early feasibility studies and technical descriptions have demonstrated that these devices can be safely applied in both minimally invasive aortic and mitral valve surgery. Recent reports have even described the successful implementation of automated suturing within fully endoscopic workflows, emphasizing its role in standardizing operative integration [18]. However, a significant gap remains in the literature. Most published studies on automated suturing focus on selected patient cohorts, single valve positions, or purely technical descriptions. Furthermore, while the term “usability” is frequently cited, it is rarely operationally defined or assessed in the context of real-world clinical practice. In a routine surgical setting, usability encompasses not only technical success but also seamless workflow integration, reproducibility across varying anatomies, and the ability to apply technology consistently without introducing new sources of error.

At the Passau Heart Center, the development of MIVS has been characterized by a decade of structured maturation [19]. Our program has documented a systematic transition from traditional open femoral cut-down to ultrasound-guided percutaneous femoral cannulation supported by advanced technologies such as the MANTA^®^ vascular closure device (Teleflex, Wayne, PA, USA) [19,20,21,22]. This “institutional evolution” has resulted in measurable improvements in procedural efficiency, including significantly shorter CPB and total operative times, as well as reduced ICU and hospital stays. Perhaps most importantly, the adoption of percutaneous techniques has virtually eliminated lymphatic morbidity, which was a common complication prior to 2021, when the groin cannulation was performed via cut-down technique [19]. Our center’s experience indicates that MIVS programs mature not through the simplification of surgery, but through the progressive acquisition of confidence in advanced techniques and the standardization of team-based workflows.

As part of this ongoing maturation, we have integrated automated annular suturing into our surgical armamentarium. While our broader MIVS program has seen an increase in the complexity of reconstructive mitral repairs—including high rates of combined annuloplasty and neochordae implantation—the RAM^®^ device has been utilized specifically for mitral and aortic valve replacements. This specialized focus allows for a unique analysis of how mechanization influences the most standardized form of valve intervention. Given that our center has already established a high-performing program for MIVS, it is essential to evaluate new technologies not in isolation, but against this modern institutional benchmark.

While automated suture fasteners have become standard adjunctive tools in MIVS, automated annular suturing devices represent a further step toward mechanizing intracardiac suture placement. However, published evidence remains limited to preliminary series and technical descriptions. Furthermore, real-world reports evaluating the initial institutional adoption, safety boundaries, and technical feasibility across both mitral and aortic valve replacement remain sparse.

In this study, “usability” is operationally understood not through formal human-factors framework testing, but through pragmatic metrics of technical success, absence of device malfunction, and operational integration into an established MIVS workflow. The primary objective is to evaluate the technical feasibility and safety of the RAM^®^ automated suturing device in consecutive minimally invasive valve replacement cases, utilizing our mature institutional percutaneous MIVS experience as descriptive contextualization.

## 2. Materials and Methods

### 2.1. Study Design, Population and Ethical Considerations

This retrospective, single-center descriptive observational study was conducted at the Passau Heart Center. The analysis focused on 43 consecutive patients undergoing minimally invasive cardiac valve surgery (MIVS) supported by the RAM^®^ automated annular suturing device. To provide institutional context, this cohort was benchmarked against the broader SoC cohort of the 5-year period (2021–2025) of the center, which included 182 patients treated with standardized ultrasound-guided groin access [20]. The study protocol for the longitudinal experience was approved by the Ethics Committee of the University of Regensburg (protocol code 25-4426-104, approval date 3 December 2025), while formal approval for the specific anonymized RAM usability analysis was waived according to local regulations.

All patients undergoing MIVS during the study period were identified via the institutional surgical database. Inclusion required undergoing isolated mitral or aortic valve replacement performed through a right-sided minimally invasive access. Routine preoperative evaluation included CT angiography to assess the suitability of femoral vessels for peripheral cannulation. Specific exclusion criteria for peripheral access included a femoral artery diameter of less than 6 mm, significant anterior calcification at the intended puncture site, or the presence of aortic thrombosis.

The primary outcome was technical success, defined as successful automated placement of all annular sutures without conversion to manual suturing or device failure. Secondary outcomes included total operative duration, cardiopulmonary bypass (CPB) and aortic cross-clamp times, device-related technical complications, 30-day mortality, postoperative valve hemodynamics, and perioperative adverse events (stroke, acute kidney injury, re-exploration for bleeding).

The institutional benchmark cohort (*n* = 182) represents the broader standard-of-care percutaneous MIVS population treated at our center across the 5-year period (2021–2025), from which the 43 consecutive RAM^®^ patients represent a distinct, prospective mechanized subcohort evaluated during its introductory phase. This benchmark cohort is presented strictly for descriptive contextualization of baseline institutional operative timeframes and recovery metrics, rather than as a formal comparative control group, given differences in case mix and anatomical pathologies.

### 2.2. Surgical Technique

**Figure 1:**
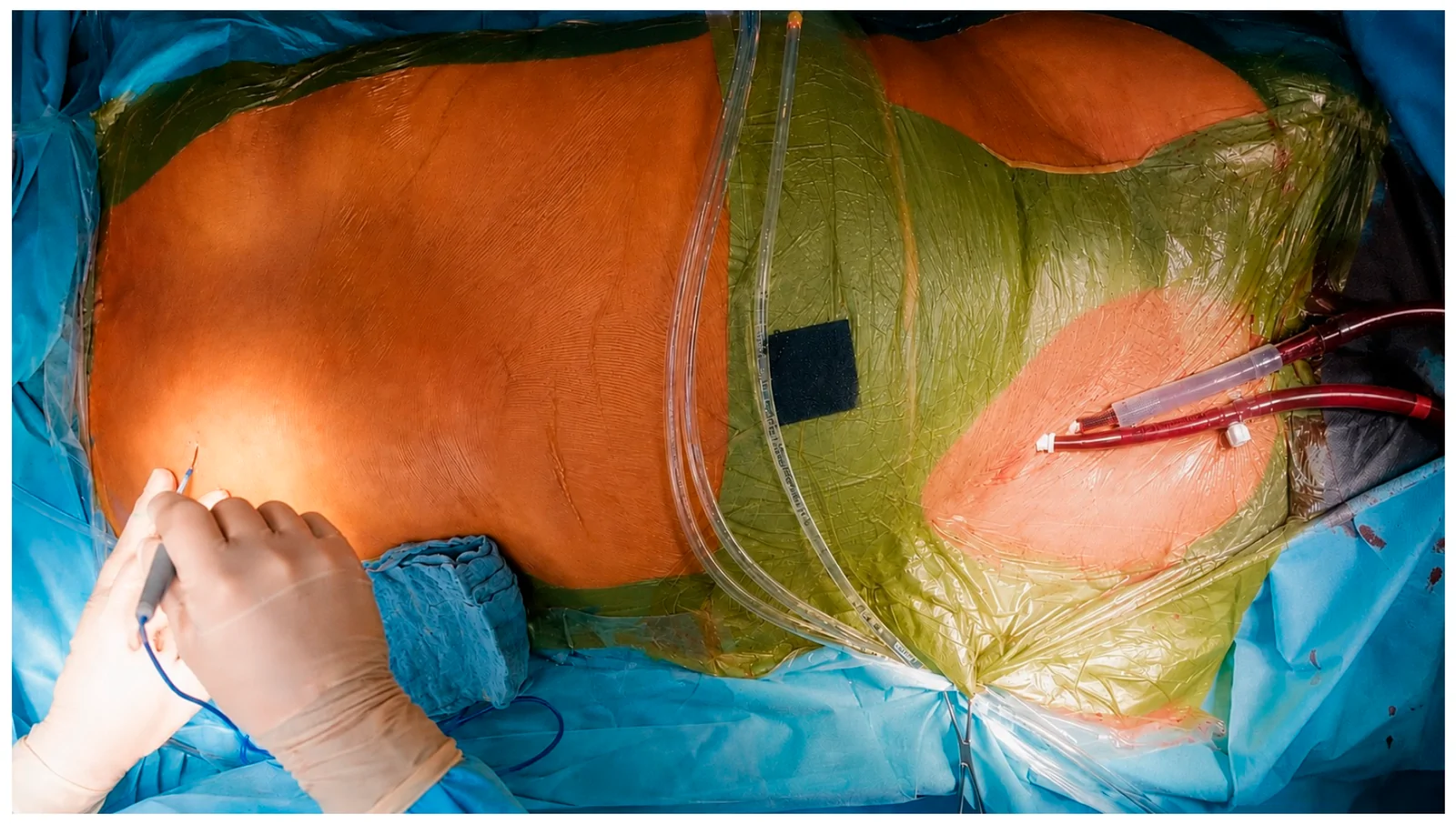
Percutaneous cannulation with the 21-Fr Bio-Medicus™ arterial cannula (Medtronic, Minneapolis, MN, USA) and a SMART Cannula^®^ for venous drainage (Smartcanula LLC, Lausanne, Switzerland).

All procedures were performed by a single experienced surgeon under general anesthesia with continuous intraoperative transesophageal echocardiographic guidance. A 4–5 cm right mini-thoracotomy in the 3rd or 4th intercostal space was used for both mitral and aortic valve procedures. Cardiopulmonary bypass was established via percutaneous femoral cannulation using a 19- or 21-Fr Bio-Medicus™ arterial cannula (Medtronic, Minneapolis, MN, USA) and a SMART Cannula^®^ for venous drainage (Smartcanula LLC, Lausanne, Switzerland) (Figure 1).

After initiation of cardiopulmonary bypass, the ascending aorta was cross-clamped using a Chitwood transthoracic clamp. Myocardial protection was achieved with a single antegrade dose of 1800 mL Bretschneider histidine–tryptophan–ketoglutarate cardioplegia (Bretschneider-HTK) administered over approximately 6 min.

Carbon dioxide insufflation of the operative field and transesophageal echocardiographic guidance were routinely used throughout the procedure. Exclusively biological prosthetic valves were implanted in all patients.

The RAM^®^ 5.0 and 3.5 automated suturing devices were deployed sequentially around the annulus as follows:

1. Device Loading and Alignment: The single-use shaft is pre-loaded with a pledgeted 2-0 braided polyester suture. Under direct endoscopic visualization, the curved device jaw is positioned across the target annular tissue segment.
2. Double-Needle Deployment: Actuating the device handle simultaneously drives two curved needles through the annular tissue from the atrial/aortic side, capturing a precise tissue bite (5.0 mm or 3.5 mm width depending on jaw selection) and pulling the suture legs back into the shaft housing.
3. Suture Retrieval & Management: The device is withdrawn through the mini-thoracotomy port, leaving the pledgeted suture anchored in the annulus. Suture limbs are organized sequentially in a circular organizer.
4. Prosthetic Threading & Fastening: Suture legs are passed through the prosthetic sewing cuff using the SEW-EASY^®^ 5.0 device (LSI Solutions, Victor, NY, USA). Following valve seat placement, all sutures are secured and trimmed flush using the CorKnot^®^ automated titanium fastener device.

#### 2.2.1. Minimally Invasive Mitral Valve Replacement

Mitral valve replacement was performed through a right anterolateral mini-thoracotomy in the fourth intercostal space. After cardioplegic arrest, the left atrium was opened through Sondergaard’s groove, and the mitral valve was exposed using a dedicated atrial retractor. The native mitral valve was excised while preserving the subvalvular apparatus whenever technically feasible.

**Figure 2:**
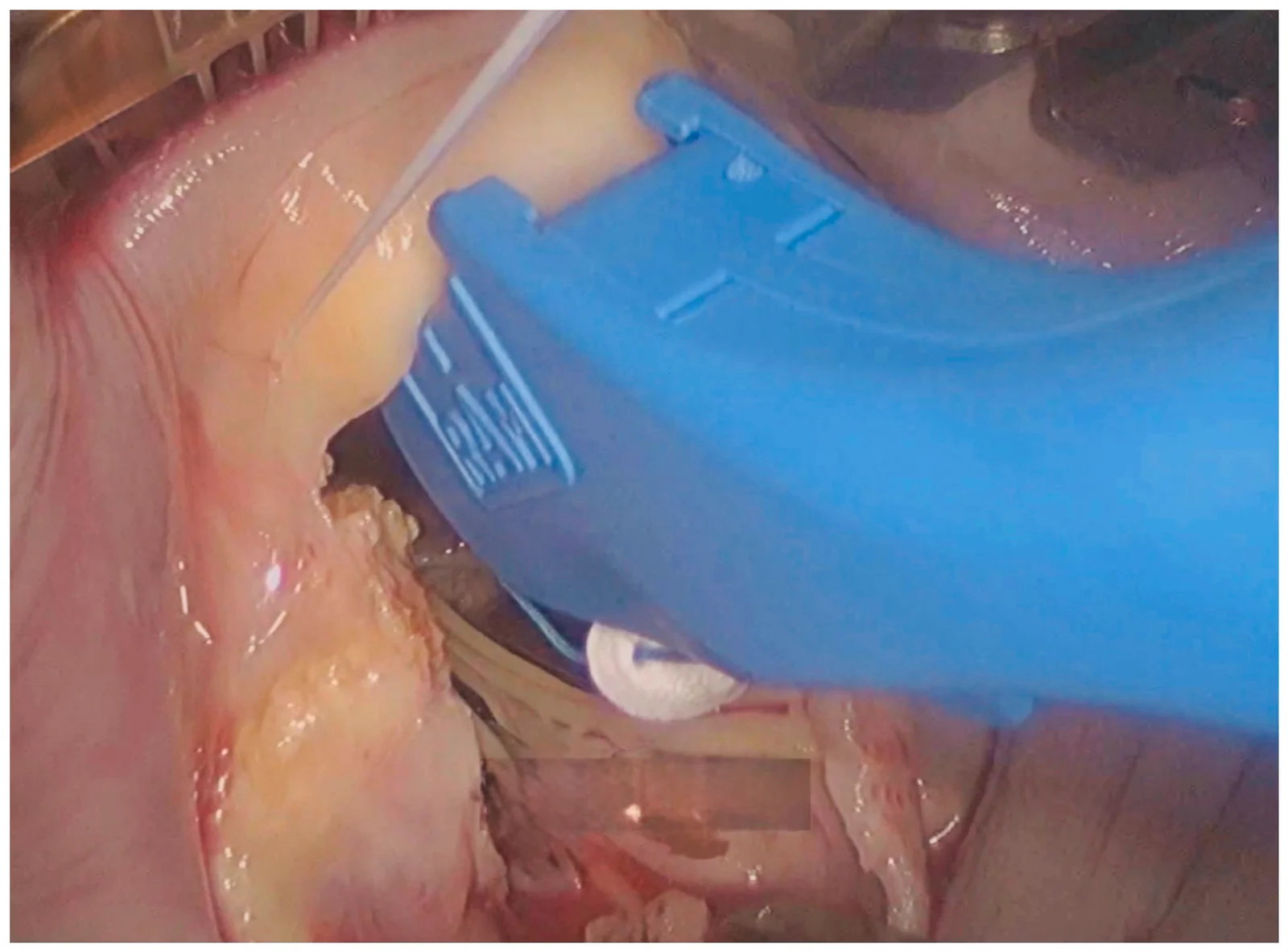
Placement of the annular sutures with the RAM 5.0 automated suturing device (LSI Solutions, Victor, NY, USA).

Interrupted pledgeted 2-0 braided polyester annular sutures were placed circumferentially around the mitral annulus using the RAM^®^ 5.0 automated suture fastening device (LSI Solutions, Victor, NY, USA) (Figure 2).

**Figure 3:**
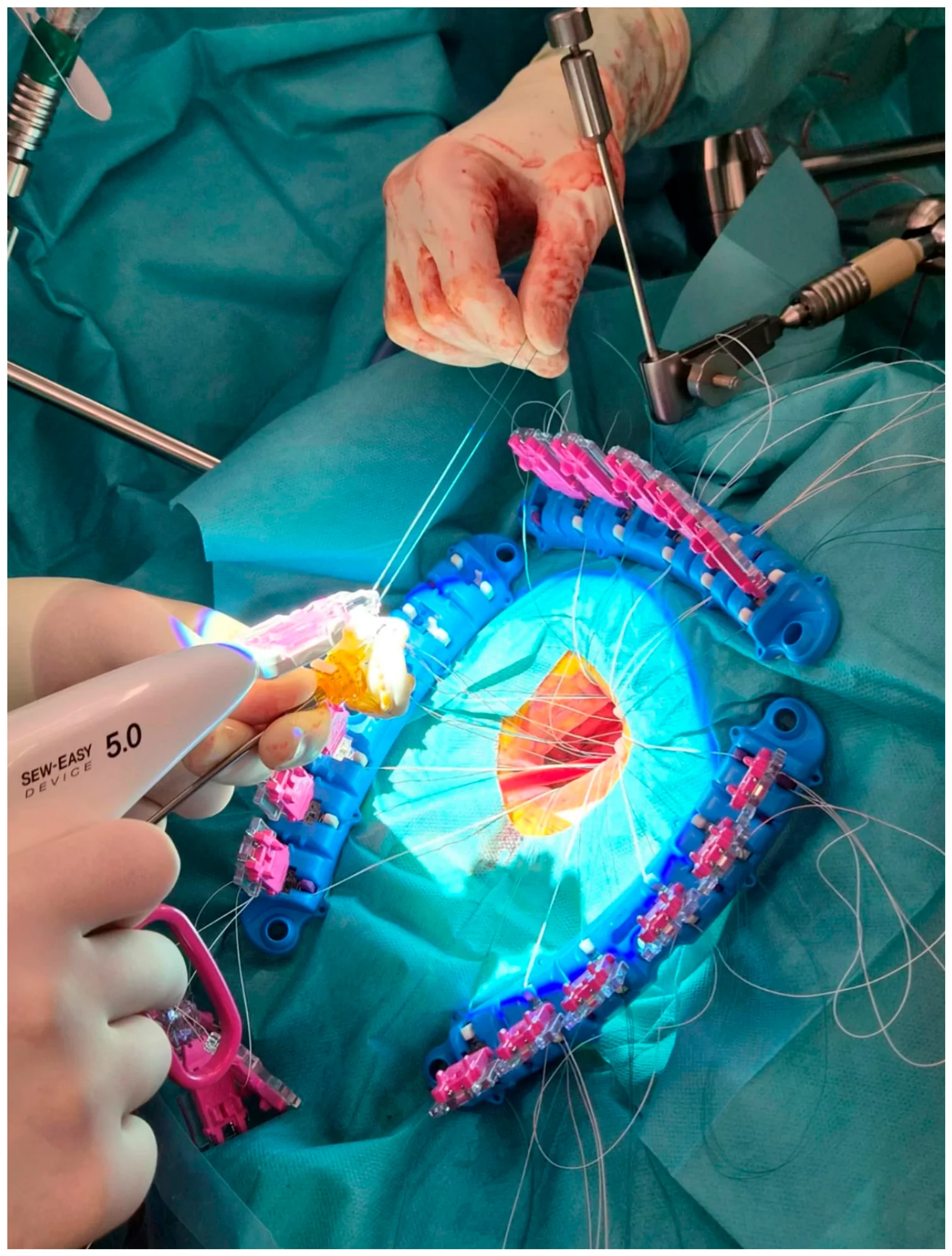
The annular sutures are passed through the prosthesis with the SEW Easy Device (LSI Solutions, Victor, NY, USA).

After placing the sutures, all sutures were passed through the prosthetic valve using the SEW-EASY Device 5.0 (LSI Solutions, Victor, NY, USA) (Figure 3).

Then the prosthesis was deployed in the mitral annulus, and the sutures were tightened with CorKnot (LSI Solutions, Victor, NY, USA). The device deploys a titanium fastener while simultaneously trimming the excess suture material, eliminating the need for manual knot tying. Following confirmation of appropriate prosthesis seating, the left atrium was closed in the standard fashion. After de-airing and removal of the aortic cross-clamp, valve function was assessed by intraoperative transesophageal echocardiography.

#### 2.2.2. Minimally Invasive Aortic Valve Replacement

Minimally invasive aortic valve replacement was performed through the same right mini-thoracotomy approach in the 2nd or 3rd intercostal space. Following cardioplegic arrest, a transverse or oblique aortotomy was performed, and the calcified native aortic valve was excised. Annular decalcification was carried out meticulously to allow optimal prosthesis implantation.

Interrupted pledgeted 2-0 braided polyester sutures were placed circumferentially around the aortic annulus using the RAM^®^ 3.5 mm (or 5.0 mm) automated suturing device. Special care was taken during annular engagement in the left- and right-coronary cusp regions, where the curved 3.5 mm jaw was oriented tangentially to ensure adequate tissue purchase while maintaining safe clearance from the coronary ostia. Suture limbs were subsequently passed through the sewing ring of the biological prosthesis using the SEW-EASY^®^ device. After seating the valve, all sutures were secured and trimmed using the CorKnot^®^ automated titanium fastening device. The aortotomy was closed in two layers in standard fashion. Following de-airing, the aortic cross-clamp was removed, and prosthetic function was verified via intraoperative transesophageal echocardiography.

### 2.3. Data Collection and Variables

Data were extracted from the institutional surgical database and reviewed manually. Variables included: Baseline characteristics: Age, sex, obesity (BMI > 30 kg/m^2^), EuroSCORE II, pre-operative NYHA class, COPD, chronic dialysis, and prior cardiac surgery (any previous commissurotomy, Harpoon, mitral valve replacement, bypass, aortic valve replacement, or prior sternotomy). Operative variables: Cardiopulmonary bypass time, aortic cross-clamp time, total operation time, ICU stay, hospital stay, and need for re-exploration for bleeding [23]. Access-site and device outcomes: Groin complications, lymph fistula, wound healing disorder, use of MANTA closure, MANTA-related complications (ischemia or bleeding). Mortality: <30-day and >30-day mortality. Groin complications were defined as a composite endpoint including access-site hematoma requiring intervention, pseudoaneurysm, arterial or venous thrombosis, limb ischemia, access-site infection, seroma, or need for surgical or endovascular vascular intervention. Lymph fistula and wound-healing disorders were analyzed separately, as lymphatic injury represents a distinct mechanism related to surgical dissection.

### 2.4. Statistical Analysis

All data were extracted from the institutional database and reviewed manually. Continuous variables demonstrated a non-normal distribution and were therefore expressed as median and interquartile range (IQR). Categorical variables were presented as absolute counts and percentages. Differences in jaw size distribution between valve positions were evaluated using Fisher’s exact test. All analyses were performed using Python 3.13 with pandas and SciPy libraries.

## 3. Results

### 3.1. Patient Population and Baseline Characteristics

The analyzed study population comprises the introductory consecutive cohort of automated annular suturing at our institution. A total of 43 patients underwent minimally invasive valve surgery (MIVS) supported by the RAM^®^ system. This mechanized cohort was stratified by anatomical position into a mitral valve replacement group (RAM-MVR, *n* = 26) and an aortic valve replacement group (RAM-AVR, *n* = 17). To contextualize these groups within our modern standardized workflow, they are presented alongside the overarching institutional standard of care (SoC) benchmark cohort (*n* = 182).

The baseline demographics and clinical profiles of the automated groups demonstrated characteristics typical of contemporary limited-access valve replacement candidates. In the RAM-MVR subgroup, the median age was 67.0 years (IQR 56.0–74.0), which was identical to the median age of 67.0 years (IQR 59.5–74.0) observed in the RAM-AVR group and closely aligned with the institutional benchmark population (median 65.0 years, IQR 57.0–74.0). Female patients accounted for 53.8% (14/26) of the mitral cohort and 17.6% (3/17) of the aortic cohort, compared to 32.4% (59/182) in the overarching institutional benchmark.

Preoperative risk scores and symptomatic severity reflected distinct clinical patterns between the two anatomical categories. The median EuroSCORE II was higher in the mitral cohort at 2.61% (IQR 1.03–4.90) than in the aortic cohort, which presented with a median score of 0.92% (IQR 0.76–1.30). Preoperative functional impairment was highly pronounced among the mitral patients, with a median NYHA functional class of 3 (IQR 2–3), and 58.1% of the RAM-MVR cohort presenting in NYHA class III or IV. The RAM-AVR cohort exhibited a descriptively lower symptomatic burden, with a median NYHA class of 2 (IQR 1–3). Preoperative left ventricular ejection fraction (LVEF) was stable and well-preserved across both active automated groups, showing a median of 56.5% (IQR 55.0–60.0%) in the mitral replacement patients and 60.0% (IQR 55.0–60.0%) in the aortic replacement patients. Baseline demographics are summarized in Table 1.

### 3.2. Operative Metrics and Procedural Efficiency

Procedural characteristics for the automated cohort demonstrated highly consistent performance profiles across both positions. For patients undergoing mitral valve replacement (RAM-MVR), the median aortic cross-clamp duration was 73.0 min (IQR 64.0–91.0), with an associated total cardiopulmonary bypass time of 114.5 min (IQR 94.8–129.5). Total surgical skin-to-skin time for the mitral cohort was 201.0 min (IQR 184.8–226.3). In comparison, the aortic subgroup (RAM-AVR) exhibited median cross-clamp and CPB times of 75.0 min (IQR 71.0–83.0) and 92.0 min (IQR 86.0–108.0), respectively, translating to a total operative duration of 171.0 min (IQR 160.0–191.0). These timeframes aligned well within the overall baseline of the institution’s mature percutaneous MIVS experience, as seen in Table 2.

### 3.3. Learning Curve and Procedural Maturation Analysis

**Figure 4:**
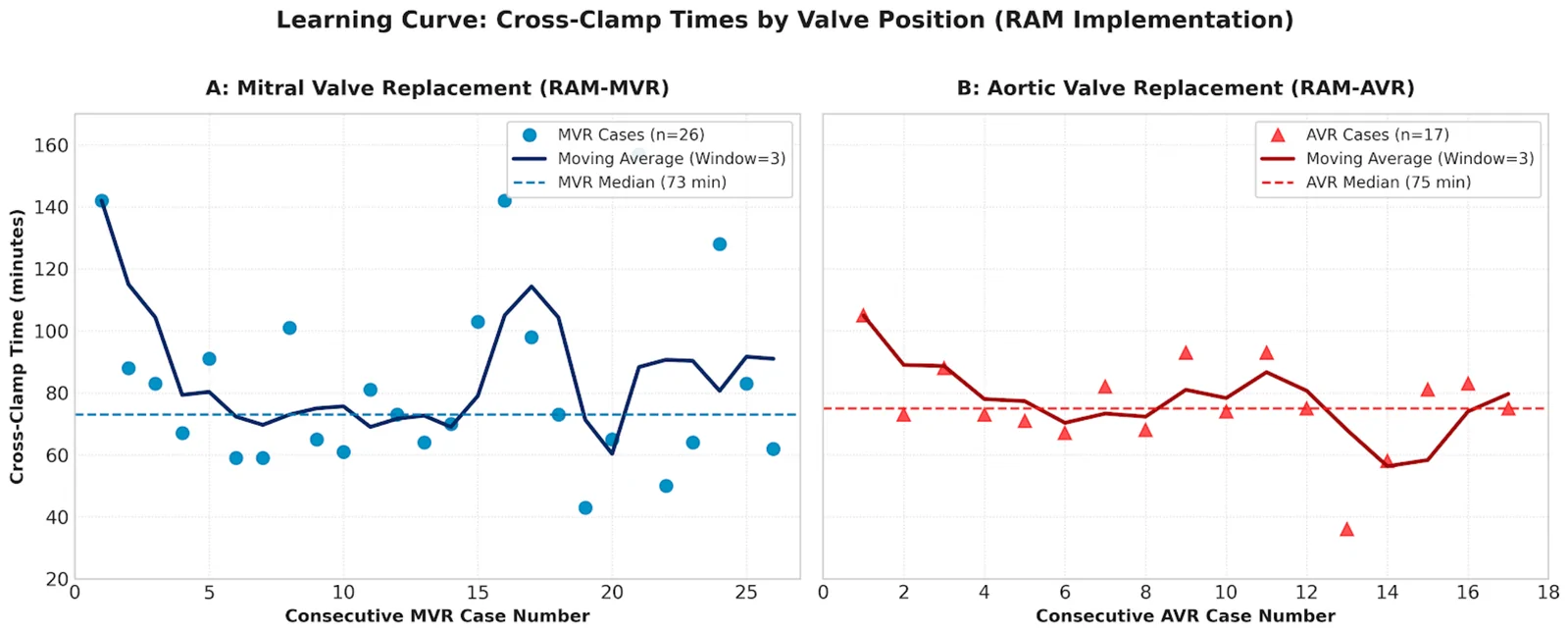
Learning Curve of Aortic Cross-Clamp Times during RAM Implementation for mitral and aortic cases.

For the RAM-MVR cohort (*n* = 26), initial cross-clamp durations frequently exceeded 90 min before stabilizing toward the subgroup median of 73.0 min (IQR: 64.0–91.0 min) after approximately 15 cases. In the RAM-AVR cohort (*n* = 17), cross-clamp times demonstrated immediate consistency from early cases, stabilizing around a subgroup median of 75.0 min (IQR: 71.0–83.0 min). Both subgroups demonstrated a reduction in overall procedural variability as surgical and nursing workflows matured (Figure 4).

### 3.4. Device Configuration and Feasibility

Technical feasibility was achieved in 100% of intended cases (43/43, 95% CI: 91.8–100.0%). No mechanical device jams, suture fraying, or conversion to conventional manual suturing were recorded (0%, 95% CI: 0.0–8.2%). Detailed jaw configurations (5.0 mm vs. 3.5 mm) for both valve positions are summarized in Table 3.

### 3.5. Postoperative Outcomes

The overall 30-day mortality in the RAM cohort was 4.7% (2/43, 95% CI: 0.6–15.8%; RAM-MVR: 3.8% [1/26], RAM-AVR: 5.9% [1/17]). Clinical review confirmed that neither event was related to device failure, suture disruption, or paravalvular leak. One RAM-MVR patient (EuroSCORE II: 4.90%, NYHA class IV) developed severe postoperative pneumonia resulting in septic shock, while one RAM-AVR patient developed severe vasoplegia and multi-organ failure (MOF). Median ICU stay was 2.0 days (IQR 1.0–3.0) for both groups, with a median total hospital stay of 10.0 days (IQR 9.0–13.3 in RAM-MVR; IQR 8.0–12.0 in RAM-AVR). Predischarge echocardiography demonstrated optimal prosthetic seating with zero instances of paravalvular regurgitation (0%, 95% CI: 0.0–8.2%) and median mean transvalvular pressure gradients of 3.0 mmHg (IQR 2.0–4.0) for mitral prostheses and 8.0 mmHg (IQR 6.0–10.0) for aortic prostheses (Table 4).

Postoperative cardiac function remained stable (median LVEF 55% across all cohorts), and recovery metrics showed no descriptive difference in ICU or hospital stay compared to the institutional benchmark.

## 4. Discussion

The integration of automated annular suturing technology into minimally invasive mitral and aortic valve surgery represents a strategic pivot toward procedural standardization at our institution. By profiling the introductory cohort of the RAM^®^ device within our mature clinical environment, the primary findings demonstrate that automated suturing is not only highly feasible but achieves an operative efficiency profile that aligns well with established institutional standards from the early adoption phase.

### 4.1. Procedural Efficiency and Workflow Integration

The clinical introduction of the RAM^®^ automated annular suturing device at our institution demonstrated highly stable perioperative metrics that integrated effectively into our standardized percutaneous MIVS program. Cross-clamp duration and overall procedural efficiency are strongly influenced by visual exposure and aortic occlusion techniques during limited-access valve procedures [23]. In limited-access cardiac surgery, the aortic cross-clamp duration is widely considered the primary indicator of intracardiac technical proficiency and surgical workflow efficiency [9,10,13] In our initial series of mechanized cases, the RAM-MVR cohort achieved a median cross-clamp time of 73.0 min (IQR 64.0–91.0). This profile indicates that the automated approach provides predictable and reproducible surgical timeframes from the early stages of its adoption curve.

Mechanizing the placement of annular sutures appears to streamline the procedural steps within the deep, restricted operative field of the mitral valve. By transitioning from manual needle handling to automated deployment, the surgical team bypasses the ergonomic challenges associated with working through limited port access [15,16]. This consistency is also reflected in the median CPB times for the RAM-MVR group (114.5 min, IQR 94.8–129.5), which aligned with the established institutional benchmark of 115.0 min (IQR 98.0–141.0), representing our mature percutaneous platform. The automated system acts as a reliable technical standardizer, stabilizing operative duration during the implementation of a new device workflow [12].

### 4.2. Optimization of Operative Efficiency and Technical Maturation

Rather than demonstrating a uniform learning curve across all applications, the institutional implementation of automated annular suturing revealed distinct, valve-position-specific maturation trajectories when stratified by anatomical location. In limited-access cardiac surgery, the aortic cross-clamp duration serves as the primary marker of intracardiac technical proficiency and team workflow efficiency.

In the mitral replacement cohort (RAM-MVR, *n* = 26), cross-clamp times followed a classic learning curve profile. Initial procedures frequently exceeded 90 to 100 min of ischemic time, reflecting the initial ergonomic adjustments required when introducing automated double-needle delivery shafts into the restricted visualization field of the left atrium. However, following an initial maturation phase of approximately 15 cases in the mitral cohort, procedural execution stabilized around a predictable subgroup median of 73.0 min (IQR: 64.0–91.0 min). This stabilization confirms that the initial operational overhead of device loading and suture management is rapidly offset by the efficiency of mechanized suture deployment once baseline familiarity is achieved. This maturation period is consistent with learning curves observed in other centers adopting minimally invasive techniques [24,25], where statistical process monitoring and CUSUM analyses are routinely utilized to evaluate surgical performance and procedural stabilization [26,27].

In contrast, the aortic replacement cohort (RAM-AVR, *n* = 17) displayed early procedural consistency without a prolonged initial learning phase, establishing a stable median cross-clamp time of 75.0 min (IQR: 71.0–83.0 min). The lower baseline variability in the aortic group is largely attributable to the direct visual axis of the aortic root through a right mini-thoracotomy and the predominant use of the smaller 3.5 mm jaw configuration, which allowed for efficient, repetitive suture deployment around the annulus without extensive spatial reorientation.

Crucially, the observed stabilization of operative times represents an institutional team maturation rather than purely individual surgical dexterity. The RAM^®^ system shifts intracardiac suturing from a solitary, surgeon-driven task into a synchronized, team-based workflow. Efficiency gains depend directly on tri-directional coordination between the surgeon, assistant, and scrub nurse. As the assistant becomes proficient in maintaining optimal suture trajectory and managing the pre-placed suture magazine outside the port space, the time interval between individual annular deployments shrinks significantly. By standardizing the most technically variable phase of minimally invasive valve replacement—annular suture placement—the automated system provides a reproducible framework that stabilizes operative timeframes within clinically safe boundaries.

### 4.3. Anatomical and Risk Considerations: Mitral vs. Aortic

The baseline analysis reveals distinct clinical and risk profiles between the two active automated cohorts. The RAM-AVR subgroup represented a low-risk surgical population with a median EuroSCORE II of 0.92% (IQR 0.76–1.30). In contrast, the mitral replacement cohort exhibited a higher risk profile, with a median EuroSCORE II of 2.61% (IQR 1.03–4.90). This descriptive variance reflects clinical reality, as patients requiring mitral valve replacement frequently present with advanced structural disease, illustrated by the 65.4% (17/26) of RAM-MVR patients presenting in severe NYHA functional class III or IV.

Despite these divergent preoperative risk profiles and the higher technical demands inherent to the mitral position, the mechanized operative metrics remained consistent across both subgroups. The device demonstrated operational versatility in adapting to these differing anatomical requirements. The mitral annulus, typically being more voluminous, required a larger tissue bite to ensure stable anchoring of the prosthetic sewing cuff, which explains the significant institutional preference for the 5.0 mm jaw configuration in 96.2% (25/26 cases) [28]. Conversely, the restricted space of the aortic root was effectively managed using the smaller 3.5 mm jaw configuration in 70.6% of documented cases (12/17), which allowed for optimized maneuverability around the coronary ostia without compromising suture lines. This split-profile utilization highlights the necessity of anatomically-specific device selection to ensure optimal clinical integration.

### 4.4. Synergy with the Institutional Benchmark

The successful implementation of automated suturing was inextricably linked to our center’s broader transition to the SoC cohort [20,23] As documented in our 10-year experience, the adoption of ultrasound-guided femoral access and the MANTA^®^ closure device revolutionized our MIVS program by eliminating lymphatic morbidity and standardizing vascular access [19,29].

By the time the RAM device was introduced, the “access trauma” and “access anxiety” had been largely resolved. This allowed the surgical team to focus exclusively on the intracardiac portion of the procedure. The RAM device fits perfectly into this philosophy of procedural standardization. Just as ultrasound guidance removed the variability of femoral access, automated suturing removes the variability of manual annular needle placement. The cumulative effect of these technologies is a more “industrialized” and reproducible surgical workflow, which is essential for the continued expansion of minimally invasive programs.

A critical facilitator for the successful rollout of the RAM^®^ device was the pre-existing institutional proficiency in a standardized percutaneous workflow. Having already transitioned through a decade-long maturation—moving from open femoral cut-down to ultrasound-guided access and routine use of the MANTA^®^ closure device—the surgical team operated within a stable procedural platform [20,21,30,31,32]. This maturation effectively removed the technical ‘noise’ and morbidity associated with vascular access, such as lymphatic complications or groin infections. Consequently, the team could dedicate its full cognitive and technical resources to the intracardiac mechanized suturing process, which likely facilitated the smooth integration of the new device without compromising operative times.

### 4.5. Technical Considerations and Usability

Pragmatic usability was confirmed by the high documentation rate (88.4%) and the fact that the device was used successfully inevery intended case. The surgeon’s ability to choose between 3.5 mm and 5.0 mm jaws allows for flexibility across different annuli. The preference for the larger jaw in mitral cases is consistent with the need to capture more robust tissue in the mitral ring compared to the often calcified or more delicate aortic annulus [29,32]. The lack of any paravalvular leaks or suture dehiscence in the RAM cohort further supports the technical reliability of mechanized annular fixation.

The implementation of automated annular suturing shifted the procedural demands from a purely surgeon-centric task to a highly coordinated ‘team-sport’ workflow. Unlike manual suturing, where the assistant primarily provides suction and suture tensioning, the RAM^®^ device requires the assistant to actively manage the device ‘magazine’ and coordinate the sequential delivery of sutures within the restricted port space. We observed that the stabilization of the learning curve was as much dependent on the assistant’s proficiency in maintaining optimal suture trajectory as it was on the surgeon’s precision in jaw placement. This synchronization is critical to prevent suture entanglement, a common pitfall in deep-seated minimally invasive mitral replacements.

In an exploratory, post hoc analysis, a distinct distribution in jaw configuration was observed between anatomical valve positions (Fisher’s exact test, *p* = 0.004). Rather than representing a pre-specified hypothesis test, this difference descriptively highlights the contrasting anatomical and mechanical requirements of the two target annuli. The mitral annulus—often dilated and compliant in the setting of severe regurgitation—requires a wider tissue bite to ensure robust prosthetic cuff anchoring, explaining the predominant reliance on the 5.0 mm jaw (96.2%, 25/26). Conversely, the restricted spatial geometry of the aortic root and the proximity of the coronary ostia favored the smaller 3.5 mm jaw profile (70.6%, 12/17), which offered superior maneuverability without compromising suture line stability [33,34].

### 4.6. Visual Field, Device Ergonomics, and Port Usability

A critical component of implementing advanced mechanization inside restricted fields is the geometric footprint of the instrument itself. Because the automated suturing device possesses a larger shaft and tip profile than a conventional long-shafted manual needle holder, concerns regarding visual clutter or camera obstruction are valid.

In our experience, however, this spatial footprint did not hinder procedural visualization. The dual-needle mechanical design allows for a single, well-defined entry into the annulus, eliminating the repetitive, multi-angle shifting required by traditional manual suturing. While the device tips necessitate a deliberate and focused camera path, the workflow transitions from an isolated, surgeon-centric movement to a highly structured team routine. The visual presence of the device is offset by the rapidity and precision of its tissue engagement, eliminating “trial-and-error” parallax errors frequently encountered under standard endoscopic visibility.

### 4.7. Mortality

Regarding early safety outcomes, a descriptive variance in 30-day mortality was observed between the RAM cohort (4.7%, 2/43, 95% CI: 0.6–15.8%) and the overarching institutional benchmark (1.6%, 3/182, 95% CI: 0.3–4.7%). Crucially, a granular chart review confirmed that neither fatality was attributable to technical device performance, suture line disruption, or valve dysfunction. One RAM-MVR patient (EuroSCORE II: 4.9%, NYHA Class IV) developed severe postoperative pneumonia culminating in septic shock, while one RAM-AVR patient experienced severe vasoplegic shock and MOF.

### 4.8. Limitations

This study has several significant limitations that must be considered when interpreting the results. First, it is a single-center, retrospective observational study with a relatively small sample size (*n* = 43), limiting statistical power for low-frequency clinical endpoints. Second, all procedures were performed by a single high-volume surgeon with substantial baseline experience in MIVS, which restricts the direct external generalizability of these findings to lower-volume centers or early MIVS learning curves. Third, the non-randomized design and the use of an unadjusted institutional benchmark cohort preclude formal comparative effectiveness conclusions or claims of procedural superiority over conventional manual suturing. Fourth, usability was inferred from procedural success rather than quantified using validated human-factors frameworks or team workload assessments. Finally, clinical outcomes are limited to the perioperative period and early discharge echocardiography; long-term follow-up is necessary to establish long-term structural valve stability, prosthetic valve endocarditis rates, and late paravalvular leaks.

## 5. Conclusions

In conclusion, this initial institutional experience demonstrates that automated annular suturing using the RAM^®^ device is technically feasible, safe, and highly reproducible in minimally invasive mitral and aortic valve replacements. The system integrates smoothly into mature percutaneous MIVS workflows without introducing device-specific morbidity, suture disruption, or mechanical failure.

Mechanization provides a standardized technical platform for intracardiac suture placement, achieving stable operative durations and preserved hemodynamic performance across varying anatomical valve positions. The pronounced anatomical preference for specific jaw sizes highlights the necessity of anatomical-tailored device selection to ensure optimal prosthetic fixation.

While these observational findings confirm consistent technical performance during early institutional adoption, prospective randomized controlled trials with expanded patient cohorts and long-term echocardiographic surveillance remain necessary to definitively evaluate comparative efficiency, cost-effectiveness, and long-term prosthetic durability.

## Figures and Tables

**Table 1:** Baseline characteristics of the patient population.

Parameter	RAM-MVR (*n* = 26); [95–CI]	RAM-AVR (*n* = 17); [95–CI]	Institutional Benchmark (*n* = 182); [95–CI]
Demographics			
Age (years) [Median (IQR)]	67.0 (56.0–74.0)	67.0 (59.5–74.0)	65.0 (57.0–74.0)
Female sex [% (*n*)]	53.8% (14/26)	17.6% (3/17)	32.4% (59/182)
Clinical Status			
EuroSCORE II (%) [Median (IQR)]	2.61 (1.03–4.90)	0.92 (0.76–1.30)	1.21 (0.73–2.51)
NYHA Class [Median (IQR)]	3 (2–3)	2 (1–3)	3 (2–3)
Echocardiography			
LVEF (%) [Median (IQR)]	56.5 (55.0–60.0)	60.0 (55.0–60.0)	N/A

**Table 2:** Surgical characteristics.

Outcome Parameter	RAM-MVR (*n* = 26)	RAM-AVR (*n* = 17)	Institutional Benchmark (*n* = 182)
Aortic X-clamp [Median (IQR)]	73.0 (64.0–91.0)	75.0 (71.0–83.0)	N/A
CPB time [Median (IQR)]	114.5 (94.8–129.5)	92.0 (86.0–108.0)	115.0 (98.0–141.0)
Total surgery time [Median (IQR)]	201.0 (184.8–226.3)	171.0 (160.0–191.0)	210.0 (185.0–245.0)
Clinical Outcomes			
30-day mortality [% (*n*)]	3.8% [1/26, 0.1–19.6%]	5.9% [1/17, 0.1–28.7%]	1.6% [3/182, 0.3–4.7%]
Recovery & Function			
ICU stay (days) [Median (IQR)]	2.0 (1.0–3.0)	2.0 (1.0–3.0)	2.0 (1.0–4.0)
Hospital stay (days) [Median (IQR)]	10.0 (9.0–13.3)	10.0 (8.0–12.0)	8.0 (7.0–12.0)
Postoperative EF (%) [Median]	55.0%	60.0%	55.0%

**Table 3:** RAM Usage.

Parameter	RAM-MVR (*n* = 26) [95–CI]	RAM-AVR (*n* = 17) [95–CI]	Institutional Benchmark (*n* = 182)
**Vascular Access & Closure**			
Percutaneous Access [% (*n*)]	100% (26)	100% (17)	100% (182)
MANTA^®^ Closure Device [% (*n*)]	100% (26)	100% (17)	100% (182)
**RAM^®^ Configuration**			
Technical Feasibility [%]	100% [91.8–100.0%]	100% [91.8–100.0%]	N/A
Jaw Size: 5.0 mm [% (*n*)]	96.2% (25)	29.4% (5)	N/A
Jaw Size: 3.5 mm [% (*n*)]	3.8% (1)	70.6% (12)	N/A
**Surgical Outcomes**			
Suture Line Failure [% (*n*)]	0% (0) [0.0–8.2%]	0% (0) [0.0–8.2%]	N/A
Paravalvular Leak [%]	0% [0.0–8.2%]	0% [0.0–8.2%]	<1%
Device Malfunction [%]	0% [0.0–8.2%]	0% [0.0–8.2%]	N/A

**Table 4:** Postoperative mean pressure gradients.

Parameter	RAM
Mean Pressure Gradient mitral valve	3.0 (2.0–4.0) mmHg
Mean Pressure Gradient aortic valve	8.0 (6.0–10.0) mmHg

## Data Availability

The data presented in this study are available from the corresponding author upon reasonable request. The data are not publicly available due to privacy and ethical restrictions.
